# Is ‘living apart together’ a real advantage for patients consulting for sexual dysfunction? A cohort study

**DOI:** 10.1111/andr.70023

**Published:** 2025-03-18

**Authors:** Clotilde Sparano, Giovanni Corona, Giulia Rastrelli, Linda Vignozzi, Daniele Vignoli, Mario Maggi

**Affiliations:** ^1^ Endocrinology Unit, ‘Mario Serio’ Department of Experimental and Clinical Biomedical Sciences University of Florence Florence Italy; ^2^ Endocrinology Unit, Azienda AUSL Maggiore Hospital Bologna Italy; ^3^ Andrology, Women's Endocrinology and Gender Incongruence Unit, ‘Mario Serio’ Department of Experimental and Clinical Biomedical Sciences University of Florence Florence Italy; ^4^ Department of Statistics, Computer Science, Applications University of Florence Florence Italy

**Keywords:** ageing, cardiovascular disease, erectile dysfunction, LAT, life style, testosterone

## Abstract

**Background:**

Quantitative research on families has introduced a new category of relationship called living apart together, that is, when a couple in a committed relationship live in separate homes. This study aimed to confirm whether living apart together improves sexual relationships in those suffering from sexual dysfunction.

**Methods:**

This study comprised cross‐sectional (*N* = 4852) and longitudinal (*N* = 1402) analyses. The former explored psychobiological, hormonal and relational correlates using the Structured Interview on Erectile Dysfunction scale. The latter assessed the occurrence of major adverse cardiovascular events in a subset of patients followed for 4.3 ± 2.59 years.

**Results:**

Compared to cohabiting couples, patients in living apart together relationships were younger and characterised by higher education levels, healthier lifestyles and lower comorbidity burdens (all *p* < 0.001). After adjusting for those confounders, the living apart together group reported better sexual functioning, more frequent sexual intercourse, and higher total testosterone levels (*p* < 0.001), and the relationship was often a source of conflicts within the familial context and of shorter duration (all *p* < 0.05). When total testosterone was included in a fully adjusted analysis of covariance, the difference in obtaining a full erection between cohabiting and living apart together relationships became non‐significant (*p* = 0.086), suggesting a hormonal influence on erectile dysfunction. In the longitudinal analysis, Cox models adjusted for the aforementioned confounders showed that living apart together relationships are associated with a twofold greater risk of major adverse cardiovascular events than cohabiting relationships, independent of other risk factors, including total testosterone levels, waist circumference and pathological penile blood flow.

**Discussion:**

This study illustrates that partnership arrangements can shape sexual interest and complaints, as reported by the participants. While men involved in living apart together relationships show an ostensibly healthier phenotype, they experienced more often major adverse cardiovascular events. Therefore, co‐residential relationship appears to provide more effective protection against future major adverse cardiovascular events for the male partner than a living apart together relationship.

## 1 INTRODUCTION

Over the past 40 years, quantitative research on families and couples has introduced a new category of relationship called living apart together (LAT). These are seen as a distinct form of intimate partnership, offering an alternative to the more traditional cohabiting relationships.^1^ Therefore, LAT relationships provide a more encompassing view of partnerships in family studies.^2^ However, studies directly comparing cohabiting and LAT relationships remain limited,^3^, ^4^ and few have explored sexual dynamics within LAT relationships. Sexuality plays a crucial role in forming and sustaining romantic relationships, with evidence suggesting that sex often strengthens relationship bonds.^5^, ^6^, ^7^ While recent research in sex and family demography has examined partnerships, the focus has primarily been on sexual frequency^8^, ^9^, ^10^ or satisfaction.^11^, ^12^ Conversely, data related to LAT relationships are lacking.

The broad use of the term LAT has obscured the diversity within these non‐cohabiting relationships, where partners may not always identify as a couple. In recent decades, approximately 10% of individuals are in LAT relationships in wealthier nations.^1^ The diversity of LAT relationships and their context‐specific nature are often tied to partners’ life events and personal histories. For example, LAT relationships may occur between young people testing emotional and sexual compatibility before moving in together, between divorced individuals, or between older adults who have experienced separation, divorce or widowhood.

The role of sex in LAT relationships is subject to competing theoretical perspectives. While research suggests that sexual fidelity is a key component of LAT relationships, forming the basis of commitment,^13^, ^14^ this may be because of the absence of shared social and economic resources typically found in cohabiting relationships.^15^ Unlike cohabiting partners who might pool incomes for mortgage or household expenses, those in LAT relationships tend to avoid these structural investments.^14^ Some LAT partners view their relationship as mutual support and affection, with a desire for long‐term commitment and a willingness to navigate difficulties together.^16^ In such cases, sexual activity may be seen as a primary resource or investment for LAT partners. However, research suggests that sex may not play a central role in LAT relationships. For example, living apart allows some partners to maintain emotional distance and control over their personal lives, particularly when partners have different lifestyles or offspring from previous relationships.^13^ LAT relationships might also require less commitment and involve less emotional and sexual intimacy than cohabiting relationships.^14^ Therefore, those in LAT relationships may experience less sexual intimacy than those in co‐residential relationships.

Few studies have compared sexual patterns across different relationship types, including LAT relationships. One notable exception is Ciritel's^17^ study, which examined how sexual intimacy (defined as emotional closeness during sex, sexual compatibility, and interest in sex with a partner) differs between cohabiting and LAT individuals in Great Britain. Using data from the British National Study of Sexual Attitudes and Lifestyles, Ciritel found that while LAT individuals reported higher levels of sexual intimacy than cohabiting individuals, they tended to be less satisfied with their relationships.

This study aims to extend the existing literature by examining how various aspects of male sexuality relate to singlehood, cohabiting relationships and LAT partnerships. By integrating insights from andrological and sociodemographic research, this study offers a new understanding of partnership dynamics. In particular, it aims to verify whether or not LAT improves sexual relationships in those suffering from sexual dysfunction and consulting for them at an andrological clinic in Italy. In a subset of the cross‐sectional database, the prevalence of major adverse cardiovascular events (MACE) was longitudinally investigated, according to the type of relationship.

## 2 METHODS

### 2.1 Cross‐sectional analysis

This study included consecutive cases of men referred to the Andrology Unit at Careggi University Hospital in Florence between 2002 and 2015. Their educational level, lifestyle (alcohol and smoking) habits, and number of children were collected through specific questions, as previously reported.^18^, ^19^, ^20^, ^21^ Their psychobiological and relational factors were assessed using the validated 13‐item Structured Interview on Erectile Dysfunction (SIEDY) questionnaire.^22^ Briefly, the SIEDY questionnaire is composed of three different scales estimating different aspects of male sexual dysfunction. Scale 1 assesses the organic domain and comprises questions 4, 13 and 15, asking about medical history, morning/nocturnal erection and ejaculate volume, respectively. Scale 2 assesses disturbances in the relationship with the primary partner and comprises questions 7, 8, 9 and 10, asking about the primary partner's disease, primary partner's climax and desire, and menopausal symptoms.^23^ Scale 3 assesses psychological traits and comprises questions 2, 3, 6, 11, 12 and 14, asking about life stressors, conflict in primary relationships and within the family, extramarital affairs and hypoactive sexual desire, respectively.^24^ The interviewer codified the patient's answers on a 0–3 Likert scale. In particular, the standard question 5 used here refers to the relationship type: ‘Do you have a stable relationship with a partner? Do you live together?’ (a stable relationship means a relationship lasting for at least two months that includes sexual intercourse). The answer was rated as follows: 0 = stable relationship, living together; 1 = stable relationship, not living together; 2 = no stable relationship.^22^ In addition, different specific SIEDY questions were used, codifying the answers as a dummy yes/no variable (‘reduced partner orgasm’, ‘reduced partner libido’, ‘partner menopause’ and ‘fatherhood’).

The presence of spontaneous erection was assessed using question 6 of the ANDROTEST^25^ (‘Do you ever wake up with an erection? How often has it happened in the last 3 months?’), rated as follows: 0 = yes, regularly; 1 = less frequently than in the past; 2 = only occasionally; and 3 = never. The frequency of masturbation was assessed using question 7 of the ANDROTEST (‘How often have you practised autoeroticism [masturbation] in the last 3 months?’). In order to enhance clarity in the presentation of the results, the score for this item was recorded as follows: 0 = never; 1 = 1–2 times/month; 2 = 3–7 times/month; and 3 = ≥8 times/month. The frequency of sexual intercourse was assessed using a standard question (‘During the last 3 months, how many sexual attempts per month did you have?’), rated as follows: 0 = none; 1 = 1–2 times/month; 2 = 3–7 times/month; and 3 = >7 times/month. Any sexual attempt (coital or non‐coital) was considered in the analysis, as previously reported.^10^

Erectile dysfunction was assessed using SIEDY Appendix A, as previously described.^22^ In particular, question 1 in Appendix A: ‘Describe what happens during sexual intercourse: do you have an erection? Is it a complete erection? Is it sufficient for penetration? How often?’, rated as follows: 0 = sometimes, 1 = quite often, 2 = often, and 3 = always.

To better explore patients’ psychological symptomatology, the Middlesex Hospital Questionnaire (MHQ), which screens potential mental disorders in a non‐psychiatric setting,^26^ was also utilised. This test explores different psychiatric domains, including free‐floating (MHQ‐A) or phobic anxiety (MHQ‐P), obsessive behaviours (MHQ‐O), somatisation (MHQ‐S) or depressive (MHQ‐D) symptoms, and histrionic personality (MHQ‐H). Notably, the total MHQ score (∑MHQ) identifies each subject's psychological attitude and personality.

Each patient underwent a complete clinical and blood test examination. The primary clinical data collected were body mass index (BMI), waist circumference (WC), testis volume and arterial blood pressure. Metabolic syndrome (MetS) factors were defined based on the criteria of the National Heart, Lung and Blood Institute/American Heart Association as the presence of the following conditions: WC > 102 cm, triglycerides > 150 mg/dL or treatment, high‐density lipoprotein‐cholesterol (HDL‐C) < 40 mg/dL or treatment, blood pressure > 130/85 mmHg or treatment, or fasting plasma glucose > 100 mg/dL or treatment.^27^ The impact of concurrent comorbidities was estimated using the chronic disease score (CDS) calculated based on the patient's medical history and drugs.^28^

Each patient underwent a full biochemical and hormonal screening. Blood samples were drawn in the morning after an overnight fast to measure glucose (hexokinase method; Dimension Vista 1500 Medical Solutions; Siemens Healthcare); glycated haemoglobin (HbA1c; high‐performance liquid chromatography, Variant II method; BioRad Laboratories); total cholesterol, HDL, and triglycerides (automatic enzymatic colourimetric method; Dimension Vista 1500 Medical Solutions; Siemens Healthcare); total testosterone (TT) and prolactin (electrochemiluminescent method; Roche); sex hormone binding globulin (electrochemiluminescence immunoassay; COBAS Molecular Analyser; Roche, https://careers.roche.com/global/en/germany); thyroid‐stimulating hormone, follicle‐stimulating hormone and luteinising hormone. Low‐density lipoprotein cholesterol (LDL‐C) was calculated using the Friedewald equation (LDL‐C = total cholesterol ‒ [HDL‐C + triglycerides/5]).

Penile colour‐Doppler ultrasound (PDCU) was performed at the Andrology Unit of Careggi Hospital in 1747 cases, and the basal peak systolic velocity (bPSV) under flaccid conditions was collected. Of these cases, 1676 also underwent a dynamic assessment after an intracavernous injection of 10 µg of prostaglandin E1 to determine the dynamic PSV (dPSV), as previously reported.^29^, ^30^ The characteristics of these subsets did not differ from those of the entire cohort (data not shown). All the data provided were collected as part of the routine clinical procedure, according to our hospital's approved protocol (L99‐A08 292/2014) for the diagnostic workup for each patient referred to our unit for sexual dysfunction.

### 2.2 Longitudinal analysis

A subgroup of 1402 patients, followed longitudinally for 4.3 ± 2.6 years, was evaluated to assess the occurrence of MACE, as previously reported.^30^, ^31^ The characteristics of this sample did not differ from those of the entire cohort. To verify the association of relationship type with cardiovascular (CV) disease, we also examined three non‐conventional CV risk factors: TT, BMI and PCDU‐assessed penile blood flow.^30^, ^31^

### 2.3 Statistical analysis

Continuous variables are expressed as the mean ± standard deviation or median (interquartile range) when normally or non‐normally distributed, respectively. Categorical variables are expressed as percentages. A Kruskal–Wallis test or analysis of variance (anova) was used to compare groups according to their sample size and distribution. Unpaired two‐sided Student's *t*‐tests were used to compare the means of normally distributed variables between groups. In all other cases, the Mann–Whitney *U*‐test was used to compare variables between groups. In the cross‐sectional analysis, fully adjusted (age, education, lifestyle and CDS) analysis of covariance (ancova) models were used to estimate the effect of relationship type (cohabiting, LAT or no stable relationship) on continuous outcomes (WC, triglycerides, total cholesterol, TT and calculated free testosterone [cFT]). Similarly, fully adjusted (age, education, lifestyle and CDS) logistic binary regression analyses were performed to verify the effect of LAT versus cohabiting relationships on categorical or ordinal outcomes (ability to penetrate, loss of spontaneous erection, frequency of masturbation, reduced sexual desire, frequency of intercourse, conflict in the family and MetS factor). In the longitudinal analysis, Kaplan–Meier curves were used to calculate survival‐free from MACE in the entire cohort and by relationship type. In addition, fully adjusted (age, education, lifestyle and CDS) Cox regression analyses were performed to assess the risk of future MACE under different models, including unconventional CV risks, such as maximal dPSV, TT and WC. All analyses were performed using spss (version 26; IBM), and all figures were produced using GraphPad Prism (version 9; GraphPad Software).

## 3 RESULTS

This study evaluated 4852 patients (mean age: 51.3 ± 13.3 years), of which 3471 (71.5%) were in a stable cohabiting relationship, 792 (16.3%) were in a stable LAT relationship and 589 (12.1%) were not in a stable relationship. Table 1 presents the demographic characteristics of the entire cohort by relationship type, highlighting differences among groups. Those reporting being in a stable cohabiting relationship were older than those in other types of relationships and often had unhealthy lifestyles and lower education levels (Table 1). They also exhibited a greater burden of comorbidities, according to the CDS (Table 1). Therefore, all subsequent analyses were adjusted for age, CDS, education and lifestyle (including smoking and drinking behaviour). Interestingly, even in this fully adjusted model, those reporting no stable relationship or a LAT relationship more frequently reported a habit of cannabis use (*p* = 0.005, data not shown).

**TABLE 1:** Demographic data of 4852 subjects consulting for sexual dysfunction, according to the relationship status.

Factor	Stable relationship— LAT (*N* = 792)	Stable relationship— non‐LAT (*N* = 3471)	No stable relationship (*N* = 589)	*p*‐value^a^
Age (years), mean (SD)	43.23 (14.72)	54.59 (11.27)	42.83 (13.92)	**<0.001**
Years of education, *n* (%)				
≤5	35 (6.3)	314 (14.6)	36 (9.7)	**<0.001**
≤8	136 (24.5)	738 (34.3)	107 (28.7)	
≤13	224 (40.4)	742 (34.4)	134 (35.9)	
>14	160 (28.8)	360 (16.7)	96 (25.7)	
Alcohol, *n* (%)				
<4 units	766 (97.7)	3307 (96.2)	564 (97.2)	0.070
>4 units	18 (2.3)	131 (3.8)	16 (2.8)	
Smoke, *n* (%)				
Yes	268 (34.9)	973 (28.5)	217 (37.5)	**<0.001**
No	742 (95.7)	3383 (99.1)	526 (93.6)	
Cannabis, *n* (%)				
≤2	21 (2.7)	23 (0.7)	19 (3.4)	**<0.001**
>2	12 (1.5)	9 (0.3)	17 (3.0)	
Children, *n* (%)				
0	501 (66.3)	670 (19.6)	334 (73.4)	**<0.001**
1	120 (15.9)	1051 (30.8)	57 (12.5)	
2	103 (13.6)	1350 (39.6)	49 (10.8)	
3	30 (4.0)	264 (7.7)	10 (2.2)	
≥4	2 (0.3)	78 (2.2)	5 (1.1)	
BMI (kg/m^2^), mean (SD)	25.44 (3.80)	26.97 (4.16)	25.54 (4.25)	**<0.001**
Waist circumference (cm), mean (SD)	94.72 (9.92)	98.78 (10.72)	95.04 (10.89)	**<0.001**
Right testis volume (mL), mean (SD)	19.34 (4.84)	19.21 (4.75)	19.11 (5.05)	0.679
Left testis volume (mL), mean (SD)	18.82 (4.77)	18.89 (4.60)	18.76 (5.06)	0.805
Systolic blood pressure (mmHg), median [IQR]	130.00 [120.00, 140.00]	135.00 [125.00, 150.00]	130.00 [120.00, 140.00]	**<0.001**
Diastolic blood pressure (mmHg), median [IQR]	80.00 [80.00, 88.00]	80.00 [80.00, 90.00]	80.00 [80.00, 85.00]	**<0.001**
Mean blood pressure (mmHg), median [IQR]	96.67 [93.33, 103.33]	100.00 [93.33, 107.33]	96.67 [93.33, 103.33]	**<0.001**
CDS, median [IQR]	0.00 [0.00, 2.00]	1.00 [0.00, 4.00]	0.00 [0.00, 2.00]	**<0.001**
Hb (g/dL), median [IQR]	14.9 [14.4, 15.53]	14.80 [14.00, 15.6]	15.35 [15.55, 16.28]	**<0.001**
Fasting glycaemia (g/L), median [IQR]	0.90 [0.83, 1.01]	0.97 [0.88, 1.11]	0.90 [0.81, 1.02]	**<0.001**
Insulin (µUI/mL), median [IQR]	8.10 [5.8, 12.75]	8.40 [5.75, 14.00]	9.20 [6.00, 16.00]	0.538
HbA1c (%), mean (SD)	6.53 (1.62)	6.90 (1.60)	6.88 (1.91)	0.051
Total cholesterol (mg/dL), mean (SD)	197.37 (42.85)	204.31 (40.27)	191.13 (37.06)	**<0.001**
HDL cholesterol (mg/dL), median [IQR]	47.00 [41.00, 54.00]	47.00 [40.00, 55.00]	47.00 [40.00, 55.25]	0.614
Triglycerides (mg/dL), median [IQR]	102.00 [76.00, 151.00]	120.00 [86.00, 171.00]	103.50 [77.00, 153.00]	**<0.001**
Total T (nmol/L), mean (SD)	17.36 (7.00)	15.04 (6.02)	17.43 (7.14)	**<0.001**
SHBG (nmol/L), median [IQR]	32.7 [24.90, 41.50]	32.15 [24.50, 44.00]	29.70 [22.30, 40.00]	**0.039**
Calculated free T (nmol/L), median [IQR]	0.34 [0.26, 0.44]	0.28 [0.21, 0.35]	0.34 [0.24, 0.44]	**<0.001**
PDCU (cm/s), median [IQR]				
Basal PSV	17.25 [13.95, 21.50]	16.00 [12.65, 19.50]	17.00 [13.36, 21.21]	**<0.001**
Maximal PSV	51.68 [38.56, 6565]	49.00 [36.00, 60.12]	52.55 [39.65, 65.30]	**<0.001**
Acceleration	2.92 [2.12, 4.04]	2.64 [1.78, 3.53]	2.88 [2.15, 4.17]	**<0.001**
LH (UI/L), median [IQR]	3.62 [2.60, 5.20]	3.80 [2.60, 5.59]	4.00 [2.80, 5.40]	0.297
FSH (UI/L), median [IQR]	4.05 [2.70, 6.50]	4.90 [3.20, 8.20]	3.80 [2.50, 5.94]	**<0.001**
PSA (ng/mL), median [IQR]	0.80 [0.51, 1.30]	0.85 [0.52, 1.46]	0.74 [0.54, 1.20]	**0.040**
PRL (mUI/L), median [IQR]	164.11 [119.00, 237.48]	149.00 [108.00, 214.00]	175.00 [124.25, 264.00]	**<0.001**
TSH (mUI/L), median [IQR]	1.50 [1.11, 2.14]	1.41 [0.99, 2.00]	1.48 [1.09, 2.05]	**<0.001**
fT4 (pmol/L), median [IQR]	13.38 [12.02, 15.50]	13.90 [11.76, 16.02]	13.99 [12.87, 16.50]	0.374
fT3 (pmol/L), median [IQR]	5.10 [4.63, 5.70]	4.99 [4.40, 5.50]	5.22 [4.62, 5.78]	0.063
MHQ total (score), median [IQR]	28.00 [19.00, 36.00]	29.00 [19.00, 38.00]	28.00 [18.00, 38.00]	0.492
MHQ‐A (score), median [IQR]	5.00 [2.00, 8.00]	5.00 [2.00, 8.00]	6.00 [2.00, 8.00]	0.288
MHQ‐D (score), median [IQR]	4.00 [2.00, 7.00]	4.00 [2.00, 7.00]	4.00 [2.00, 7.00]	0.451
MHQ‐E (score), median [IQR]	5.00 [3.00, 7.00]	5.00 [2.00, 7.00]	4.00 [2.00, 7.00]	**0.002**
MHQ‐P (score), median [IQR]	4.00 [2.00, 5.00]	4.00 [2.00, 6.00]	4.00 [2.00, 6.00]	**<0.001**
MHQ‐O (score), median [IQR]	5.00 [3.00, 8.00]	5.00 [2.00, 9.00]	5.00 [2.00, 8.00]	0.199
MHQ‐S (score), median [IQR]	3.00 [1.00, 5.00]	3.00 [1.00, 5.00]	3.00 [1.00, 5.00]	**0.001**

*Note*: Bold numbers highlight significant comparisons.

Abbreviations: BMI, body mass index; CDS, chronic disease score; FSH, follicle‐stimulating hormone; fT3, free triiodothyronine; fT4, free thyroxine; HDL, density lipoprotein; IQR, interquartile range; LAT, living apart together; LH, luteinising hormone; max, maximum; MHQ, mental health questionnaire; min, minimum; *n*, number; PDCU, penile colour‐Doppler ultrasound; PRL, prolactin; PSA, prostatic‐specific antigen; PSV, peak systolic velocity; SHBG, sex hormone binding globulin; SD, standard deviation; T, testosterone; TSH, thyroid‐stimulating hormone.

^a^ *p*‐values refer to comparisons among the three groups.

**FIGURE 1:**
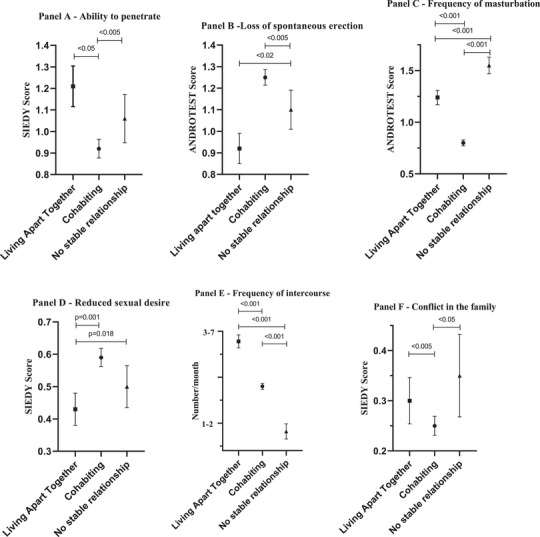
Age‐, education‐, lifestyle‐ (smoking and drinking behaviour) and chronic disease score (CDS)‐adjusted ancova model comparing sexual and relational parameters between living apart together (LAT), cohabiting and no stable relationships. The ability to penetrate, the reduced sexual desire and the conflict within the family were coded according to the SIEDY score.^22^ Loss of spontaneous erection and frequency of masturbation were coded according to the ANDROTEST score.^25^ The frequency of intercourse was coded according to a standard question (see Section 2).

Figure 1 shows the sexual patterns in the three groups. Overall, LAT relationships were characterised by a less severe sexual dysfunction than cohabiting relationships. Indeed, among patients consulting for sexual dysfunction, those reporting a cohabiting relationship showed a lower ability to penetrate (Figure 1A) and a lower frequency of spontaneous erection (Figure 1B) than the rest of the cohort. They also reported autoeroticism less often (Figure 1C). In addition, those reporting a LAT relationship showed greater sexual desire than the rest of the cohort (Figure 1D), reporting more frequent sexual intercourse (Figure 1E). However, a LAT relationship was more often a source of conflictual relationships within the familial context than a cohabiting relationship (Figure 1F). Table 1 also shows significant differences among groups in penile blood flow parameters, as assessed by PDCU. However, in the fully adjusted ancova model, those differences became non‐significant (data not shown).

When the LAT group was divided by relationship length, those in LAT relationships still showed a better ability to have an erection able to penetrate the partner than those in cohabiting relationships, but only for those in relationships with shorter durations (<5 years, *p* = 0.017), not for longer durations. When the entire cohort was divided by age tertile, those in LAT relationships reported better erections than those in cohabiting relationships but only among middle‐aged men (46–48 years, *p* = 0.016), not younger or older men. Even among these middle‐aged men, a better erection was only evident among those in LAT relationships with shorter durations (<5 years, *p* = 0.008).

In the univariate analysis, patients in cohabiting relationships reported more extramarital affairs than their LAT counterparts (17.5 vs. 12.6, *p* = 0.003, respectively). However, this difference became insignificant after adjusting for the covariates mentioned above (*p* = 0.773).

**FIGURE 2:**
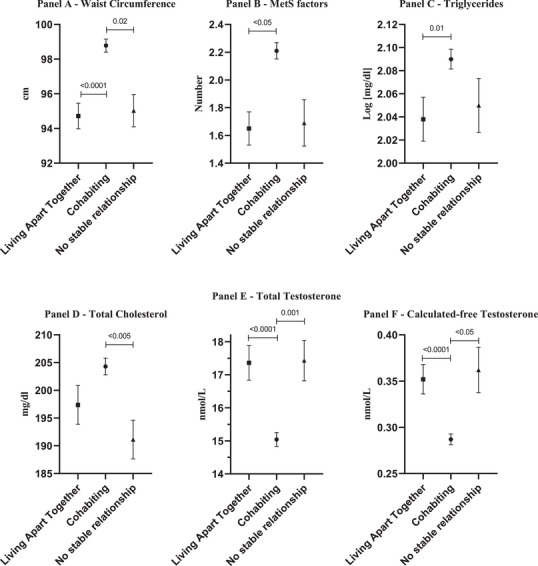
Age‐, education‐, lifestyle‐ (smoking and drinking behaviour) and CDS‐adjusted ANCOVA model comparing metabolic (A‐D) and hormonal (E and F) parameters between LAT, cohabiting and no stable relationships.

Figure 2 shows phenotypic, biochemical and hormonal parameters stratified by relationship type. In the fully adjusted model, the LAT group shows an overall healthier phenotype, characterised by an almost 5 cm smaller WC (Figure 2A) and lower accumulation of MetS factors (Figure 2B). Indeed, triglyceride and total cholesterol levels were lower in the LAT group than in the cohabiting and no‐stable relationship groups (Figure 2C,D). TT and cFT (when available) were higher in the LAT group than in the cohabiting and no‐stable relationship groups. Introducing WC into the model as a further covariate did not attenuate the significance of the differences (data not shown). When TT was introduced into the fully‐adjusted ancova model for the relationship between the ability to obtain an erection able to penetrate the partner and the relationship type, the difference between the LAT and cohabiting groups became non‐significant (*p *= 0.086), suggesting that reduced TT levels in the cohabitant group are partially responsible for the greater inability to penetrate reported by those in cohabiting relationships.

**FIGURE 3:**
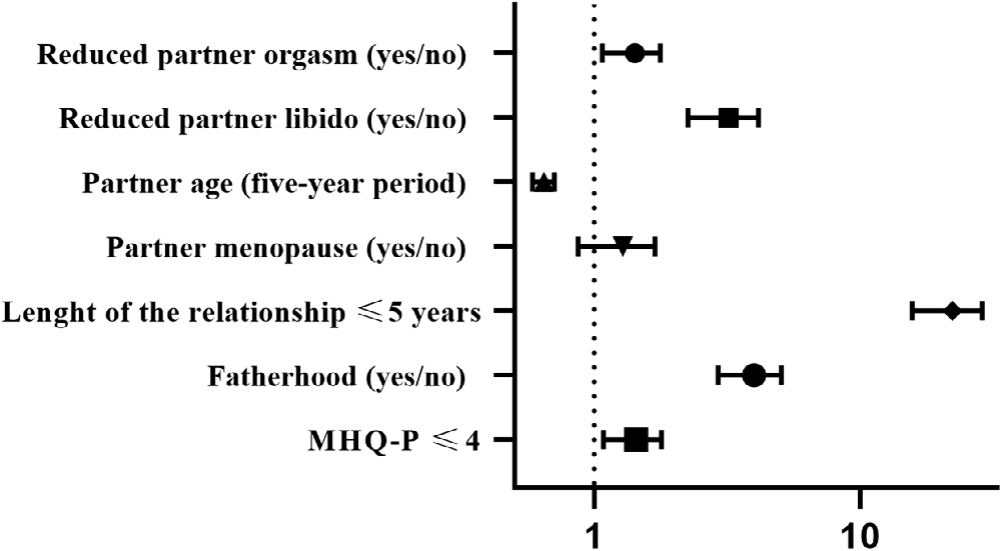
Odds ratios for relationship characteristics in iterative age‐, education‐, lifestyle‐ (smoking and drinking behaviour) and chronic disease score (CDS)‐adjusted binary regression models according to living apart together (LAT) versus cohabiting relationships (reference). The results for phobic anxiety (MHQ‐P) are also shown in the same model.

Figure 3 shows the results from fully adjusted binary logistic regressions (on a log scale) between cohabitation and LAT relationships and several relational and psychological parameters. A LAT relationship was associated with almost fourfold lower odds of fatherhood and 1.5‐fold lower odds of having a 5‐year younger partner than a cohabiting relationship. However, a positive menopausal status in the partner was only numerically higher among those in a cohabiting relationship. In addition, a LAT relationship was associated with 3.1‐ and 1.4‐fold lower odds of a partner losing sexual desire and an inability to reach climax, respectively, than a cohabiting relationship. The odds of having a relationship lasting longer than 5 years were almost 22‐fold lower among those in a LAT relationship than those in a cohabiting relationship. Finally, a LAT relationship was associated with 1.4‐fold greater odds of scoring below the median for the MHQ phobic subdomain than a cohabiting. The other MHQ subdomain differences (Table 1) were no longer significant in the fully adjusted model.

In a subset of the previous cohort followed longitudinally for a median of >4 years, we investigated whether or not the relationship type affected the risk of future MACE. No significant differences were observed among groups in a univariate Kaplan–Meier analysis. However, after adjusting for the aforementioned confounders, those in a LAT relationship had a twofold greater risk of a future MACE than those in a cohabiting relationship (Table 2). The iterative introduction of other known risk factors for this group, including TT levels, WC and pathological penile blood flow, did not significantly alter the increase in risk (Table 2).

**TABLE 2:** Age‐, education‐, chronic disease score (CDS)‐ and lifestyle‐adjusted Cox regression analyses of the relationship between living apart together (LAT) and forthcoming major adverse cardiovascular events (MACE) (model 1). In model 2 total testosterone was introduced in model 1 as a further covariate. In model 3 waist circumference was introduced in model 2 as a further covariate. In model 4 maximal dynamic peak systolic velocity at PCDU was introduced in model 3 as a further covariate.

	Model 1			Model 2			Model 3			Model 4		
	HR	95% CI	*p*‐value	HR	95% CI	*p*‐value	HR	95% CI	*p*‐value	HR	95% CI	*p*‐value
Male age	1.073	1.044‒1.102	**<0.001**	1.077	1.045‒1.109	**<0.0001**	1.081	1.047‒1.116	**<0.0001**	1.086	1.041‒1.134	**<0.0001**
Education	0.789	0.618‒1.006	0.056	0.776	0.596‒1.011	0.060	0.815	0.622‒1.069	0.140	0.797	0.567‒1.122	0.193
Smoke	1.629	0.968‒2.741	0.066	1.742	1.003‒3.026	**0.049**	1.922	1.081‒3.420	**0.026**	2.140	1.007‒4.549	**0.048**
Alcohol	1.325	0.929‒1.890	0.120	1.398	0.972‒2.010	0.071	1.396	0.962‒2.026	0.079	1.630	1.072‒2.478	**0.022**
CDS	1.161	1.055‒1.278	**0.002**	1.128	1.017‒1.252	**0.022**	1.095	0.984‒1.219	0.095	1.023	0.893‒1.172	0.741
Stable relationship—LAT	2.051	1.081‒3.892	**0.028**	2.051	1.035‒4.063	**0.039**	2.193	1.064‒4.520	**0.033**	2.654	1.176‒5.990	**0.019**
Total testosterone	**–**	**–**	**–**	1.002	0.960‒1.045	0.940	1.012	0.967‒1.059	0.610	1.030	0.972‒1.092	0.314
Waist circumference	**–**	**–**	**–**	**–**	**–**	**–**	1.056	0.994‒1.123	0.080	1.105	1.016‒1.202	**0.020**
Maximal dynamic peak systolic velocity	**–**	**–**	**–**	**–**	**–**	**–**	**–**	**–**	**–**	1.068	0.248‒4.601	0.930

*Note*: Bold numbers highlight significant comparisons.

Abbreviations: CI, confidence interval; HR, hazard ratio.

## 4 DISCUSSION

Our study demonstrated that relationship type can shape self‐reported sexual interest and complaints in a large cohort of patients complaining of sexual dysfunction. It essentially confirmed Ciritel's observation in the general population of Great Britain that individuals in LAT relationships enjoy overall greater sexual intimacy than those in cohabiting relationships.^17^

In our cohort, 16.3% of men reported being in a LAT relationship, 71.5% in a cohabiting relationship and 12.1% in no stable relationship. The proportion in a LAT relationship is slightly higher than previously reported in older studies of general populations worldwide, which indicated around 10%.^1^ However, a recent survey highlighted a notable increase in LAT relationships in a Latin American country.^32^ The reasons for this increase may be varied. In adulthood, increasing life expectancy and higher rates of relationship breakdown result in individuals experiencing more romantic relationships over time. When entering a new relationship, partners often choose to live separately before deciding to cohabit. This period of separation can be longer after a previous breakup, reflecting greater caution, a fear of repeating past mistakes,^33^ and a need to prioritise the well‐being of children.^34^ In later life, losing a partner may also influence the decision to live apart after starting a new relationship.^35^ Moreover, societal shifts have made LAT relationships more accessible, facilitated by improvements in communication and transportation, as well as a growing acceptance of unconventional family structures.

Patients in LAT relationships reported more sexual interest, more frequent sexual intercourse, and a less severe reduction of spontaneous and sexual‐related erections than those in cohabiting relationships or not in a stable relationship, in partial agreement with that observed in the general population. This finding aligns with a prior population‐level study.^17^ The frequency of autoeroticism was greater among those in LAT relationships than among those in cohabiting relationships but lower than in those not in a stable relationship. Therefore, all aspects of male sexuality are in some way more favourable among those in LAT relationships than among those in cohabiting relationships or no stable relationship. As shown in a prior study,^36^ men in LAT relationships reported a higher frequency of masturbation than those in cohabiting relationships.

Since patients in cohabiting relationships were characterised by an overall unhealthy phenotype, a greater impairment in erection is unsurprising. Indeed, those in cohabiting relationships more frequently show dyslipidaemia and increased visceral adiposity, likely because those in a stable cohabiting relationship are less prone to paying attention to their body fitness with consequent detrimental behaviours, such as overeating and sedentariness. These attitudes may lead to metabolic derangements and MetS, as reported in our study. In addition, fatherhood is more common in cohabiting relationships than in LAT relationships; it has been previously suggested that metabolic derangements increase stepwise with the number of children.^37^ However, objective parameters related to penile erection, such as those derived from penile blood flow measurements, did not differ significantly between partners cohabiting or not, suggesting that differences in organic factors do not fully explain the better sexual outcomes observed in LAT relationships.

Erectile dysfunction is not only caused by organic derangements but also by relational, hormonal and intrapsychic factors.^22^, ^38^, ^39^ Therefore, we investigated these aspects by relationship type. Patients in LAT relationships more frequently reported being involved in shorter relationships, having a younger partner with active sexual desire and being able to reach climax than those in cohabiting relationships. It was previously shown that these positive relational factors, which characterise LAT relationships, may significantly affect male sexual complaints, including issues with sexual desire, erections and intercourse frequency.^40^ In addition, the sexual fitness of the relationship is often associated with a higher TT level in the male partner,^40^ as reported in our study. Interestingly, introducing TT level as an additional covariate in the ancova model consistently attenuated the significance of the association between LAT relationship and reported erection. Therefore, higher TT levels might partially explain the better male sexual functioning in LAT relationships. We previously demonstrated that an unsatisfactory sexual relationship, as assessed by scale 2 of the SIEDY,^22^ is often associated with mild secondary hypogonadism in a bidirectional manner.^40^ Indeed, a partner's decreased sexual activity might harm the hypothalamus‒pituitary‒testis axis, and a reduced TT level might adversely affect that partner's sexual activity.^40^

Notably, the psychological burden of a stable cohabiting relationship is not comparable to that of a LAT relationship. For example, cohabiting couples more frequently manage shared financial, family (e.g., raising children), and housing responsibilities than those in non‐cohabiting relationships.^15^ Consistent with this, we observed greater levels of phobic traits among patients in cohabiting relationships than among those in LAT relationships, likely because of their fewer commitments involved beyond the sexual relationship itself.^17^ Notably, phobic anxiety is a significant psychological factor associated with erectile dysfunction.^41^ Therefore, the better sexual functioning observed in men in LAT relationships may be attributed to more favourable hormonal, relational and intrapsychic factors.

While men involved in LAT relationships showed an ostensibly healthier phenotype, with higher TT levels and lower phobic traits, we found that they more frequently experienced MACE than those in cohabiting relationships. Manfredini et al.^42^ reviewed 35 papers on MACE and marital status, finding that married men had better outcomes than single or widowed men. Similar results were recently reported in the Canadian Longitudinal Study of Aging: men (but not women) who were continuously married were more likely to age successfully than those who were never married, divorced or widowed.^43^ It is conceivable that men in a stable cohabiting relationship might exhibit greater compliance with medical controls, medication use and screening programs.^42^ The best‐documented example of the effect of marital relations on health is the so‐called ‘widowhood effect’: after the death of a spouse, men face a significantly increased risk of dying within the next few months.^44^ This effect is relatively sex‐specific, being more apparent in widowed men than in widowed women.^44^ It is particularly pronounced for CV diseases.^45^ The relevant role of a female partner in shaping the increased male CV risk has been previously emphasised.^45^ No data are currently available to compare CV risk between LAT and cohabiting relationships. However, it is plausible that the health oversight of a female partner on unhealthy behaviours in males is less stringent in non‐cohabiting relationships than in cohabiting relationships. For example, in our findings, men in LAT relationships were more likely to smoke and use illicit drugs than those in cohabiting relationships. Additionally, LAT relationships tend to be less accepted within the broader family context, which may make them more challenging.

Our study had several limitations that should be acknowledged. First, all partner‐related data were derived from patient interviews and thus reflect the male partner's perception, which, while subjective, is psychologically relevant. Second, its data were drawn from a large cohort of patients reporting sexual dysfunction, so its findings may not be generalisable to those without such conditions or to the general population. Third, its retrospective design also precludes establishing cause‐and‐effect relationships. Nonetheless, all analyses were adjusted for many major confounding variables. The prevalence of sexually transmitted infections was not available in the database. We also recognise that the sample size of the three groups varies widely. This may be a further limit for the statistical analysis performed in the study. Finally, among cohabiting couples, we could not distinguish between those who are married and those who are not, even though sexual patterns may also differ based on marital status.

In conclusion, healthcare practitioners caring for male subjects consulting for sexual dysfunction should take particular note of their type of partnership arrangement.^23^, ^40^ In particular, our study highlights the relevance of investigating relationship stability and cohabitation. We found that one in six patients reports being in a LAT relationship, a dynamic that has been rarely explored in depth in the context of sexual intimacy.^17^ Overall, LAT relationships are associated with greater sexual well‐being than cohabiting relationships. However, a stable cohabiting relationship appears to offer greater protection against future MACE in the male partner,^45^ likely because of the more continuous and stringent health oversight by the female partner.

## AUTHOR CONTRIBUTIONS

Mario Maggi conceived the study. Mario Maggi and Clotilde Sparano performed the statistical analyses. Clotilde Sparano, Giulia Rastrelli, Giovanni Corona, Linda Vignozzi, Daniele Vignoli and Mario Maggi drafted and approved the manuscript.

## CONFLICT OF INTEREST STATEMENT

The authors declare they have no conflicts of interest.

## Data Availability

Data are available from the corresponding author (M.M.) upon reasonable request.
